# Resistance and virulence genes characteristic of a South Asia Clade (I) *Candida auris* strain isolated from blood in Beijing

**DOI:** 10.1016/j.clinsp.2024.100497

**Published:** 2024-09-15

**Authors:** Jing-Xian Yang, Guan-Nan Ma, Ya-Tong Li, Yu-Peng Shi, Guo-Wei Liang

**Affiliations:** a Department of Clinical Laboratory, Aerospace Center Hospital, Beijing, 100049 China; b Zhejiang Key Laboratory of Digital Technology in Medical Diagnostics, Hangzhou, 310000 China

**Keywords:** *Candida auris*, Fluconazole resistance, Genomic, Molecular docking, Virulence

## Abstract

**Introduction:**

*Candida auris* is a globally disseminated invasive ascomycetous yeast, that imposes a substantial burden on healthcare systems. It has been documented to have spread to over 40 countries across six continents, necessitating in-depth comprehension through advanced techniques like Whole-Genome Sequencing.

**Method:**

This study entailed the isolation and Whole-Genome Sequencing of a fluconazole-resistant *C. auris* strain (CA01) obtained from a patient's blood in Beijing. Genome analysis was conducted to classify the strain, and molecular docking was performed to understand the impact of mutations on drug resistance.

**Results:**

Genome analysis revealed that CA01 belongs to the South Asia Clade (I) and shares the closest genetic relationship with previously reported strains BJCA001 and BJCA002. Notably, unlike BJCA001, CA01 exhibits significant resistance to fluconazole primarily due to the A395T mutation in the ERG11 gene. Molecular docking studies demonstrated that this mutation leads to geometric changes in the active site where fluconazole binds, resulting in decreased binding affinity. Additionally, the present findings have identified several core virulence genes in *C. auris*, such as RBF1.

**Discussion:**

The findings from this study expand the understanding of the genetic diversity and adaptive mechanisms of *C. auris* within the South Asia Clade (I). The observed fluconazole resistance driven by the ERG11 mutation A395T highlights the need for heightened awareness and adaptation in clinical treatment strategies in China. This study provides critical insights into drug resistance and virulence profiles at a genetic level, which could guide future therapeutic and management strategies for *C. auris* infections.

## Highlights

• Fluconazole-resistant *C. auris* strain (CA01) identified in Beijing, belonging to South Asia Clade (I).
• Molecular docking shows A395T mutation alters fluconazole binding in CA01. A395T mutation in ERG11 gene of CA01 linked to significant fluconazole resistance.
• Identification of core virulence genes, including RBF1, in *C. auris* strain.

## Introduction

*Candida auris* is a globally emerging fungal infection characterized by high transmissibility, multidrug resistance, and poor outcomes, with mortality rates as high as 60%.^1^ *C. auris* is less related to superficial skin and mucous membrane infections than the other species. Over the last decade, the number of reported cases has increased exponentially worldwide, and *C. auris* has been isolated in over 40 countries on six continents.^2^ In China, most reports were associated with superficial tissues, such as the urinary tract, while blood or deep tissue and organ infections were observed less frequently. However, the pathophysiology of *C. auris* infections and the molecular mechanisms underlying its drug resistance remain poorly understood, presenting therapeutic challenges for physicians.

*C. auris* is not easily recognized and identified in clinical microbiology laboratories. Currently, Whole-Genome Sequencing (WGS), an analytical technology increasingly used for sequencing the whole genomic content of bacteria, has become the new gold standard for analyzing bacterial isolates.^3^ WGS-based phylogenetic analysis divided *C. auris* into five groups, namely, clade I (South Asia), clade II (East Asia), clade III (Africa), clade IV (South America), and clade V (Iran).^4^ In mainland China, researchers isolated the first *C. auris*, named BJCA001, from the Bronchoalveolar Lavage Fluid (BALF) of the patient in 2018.^5^ After the discovery of BJCA001, another fungus, BJCA002, was discovered, with mutations in ERG11, CDR1, MDR1, and TAC1.^6^ There are at least two distinct genetic clades of *C. auris* (South Asian and South African) present in China.^7^ *C. auris*, often called superbugs, were even resistant to all three major classes of antifungal agents, including azoles, polyenes, and echinocandins. Each branch has different morphological, physiological, and genetic characteristics, such as filamentous forms, antifungal susceptibility and antifungal resistance genes. For example, mutations in the ERG11 gene are related to fluconazole resistance in *C. auris*.^8^ In addition, the virulence of *C. auris* was associated with pathogenicity, which is related to the synthesis of molecules such as phospholipases, aspartic proteases, and morphogenesis.^9^ However, both clinical and basic studies of *C. auris* are limited to date, and many open questions remain to be addressed.

In the present study, the authors report a fluconazole-resistant isolate of *C. auris* from a hospital in Beijing. This strain was isolated from blood in an intensive care patient. The authors investigate the genetic diversity and reveal the unique characteristics and multidrug resistance of this strain. Through molecular docking analysis, the authors sought to elucidate the mechanisms underlying clinically derived mutations in the ERG11 gene responsible for fluconazole resistance in *C. auris*. A comprehensive genome analysis and resistance studies demonstrated many biological and genomic differences between the newly isolated fluconazole-resistant strain and the previously reported strains. These results provide a scientific basis for clinical diagnosis and treatment and contribute to the global effort to address emerging fungal threats.

## Materials and methods

In this study, the authors reported the isolation of a fluconazole-resistant *C. auris* strain (CA01) isolated from patient's blood in Beijing and conduct genomic sequencing, resistance testing, and related resistance mechanism and virulence analysis. To ensure the rigor and transparency of the study design and reporting, the authors adhered to the STROBE guidelines.

### Strains, culture conditions and identifications

In August 2018, a strain of *C. auris* was isolated from the blood of a patient in Beijing. This strain was obtained from an 87-year-old man hospitalized in the geriatric department of a tertiary hospital. The patient was diagnosed with respiratory failure and lung infection. *C. auris* strain was routinely cultured on the chromogenic medium CHROMagar *Candida* (SHANGHAI COMAGAL MICROBIAL TECHNOLOGY CO., LDT, China) at 37°C for 48h and showed white and pink tones. Vitek MS (BioMerieux, France) was used for identification, one single colony was directly deposited on the target plate using formic acid. Both RUO (library version 4.14) and IVD (library version 3.2) were simultaneously obtained, with confidence values of 89.9% and 99%, respectively. A gold standard for identification based on ITS or D1/D2 sequences has also been performed to confirm the identification.

### Whole-Genome Sequencing, genomic assembly and functional comparative analysis

Genomic DNA was extracted with the SDS method.^10^ Libraries for Single-Molecule Real-Time (SMRT) sequencing were constructed with an insert size of 20 kb using the SMRT bell TM Template kit, version 1.0. Briefly, the process involved fragmenting and concentrating DNA, repairing DNA damage and ends, preparing blunt ligation reactions, purifying SMRTbell Templates with 0.45X AMPure PB Beads, size selection using the BluePippin System, and repairing DNA damage after size selection. Finally, the library quality was assessed on a Qubit® 2.0 Fluorometer (Thermo Scientific), and the insert fragment size was detected by Agilent 2100 (Agilent Technologies). The whole genome of CA01 was sequenced using the PacBio Sequel platform.

The genome was assembled de novo with SMRT Link v5.0.1, and gene prediction and annotation were performed with a funannotate pipeline.^11^ The sample information of the strain has been submitted to the NCBI (National Center for Biotechnology Information) database with the accession number PRJNA972483.

The authors used seven databases to predict gene functions. They were the respective GO (Gene Ontology), KEGG (Kyoto Encyclopedia of Genes and Genomes), KOG (Clusters of Orthologous Groups), NR (Nonredundant Protein Database databases), TCDB (Transporter Classification Database), P450, and Swiss-Prot. A whole-genome BLAST search (E-value less than 1e-5, minimal alignment length percentage larger than 40%) was performed against the above databases. The authors analyzed pathogenicity and drug resistance. The authors used the PHI (Pathogen Host Interactions) and DFVF (database of fungal virulence factors) to perform the above analyses.

The comparative genomic analysis included genomic synteny, core genes and specific genes. Genomic alignment between the CA01 genome and reference genome (BJCA001 and BJCA002) was performed using Mauve software.^12^ Orthologous gene clusters were compared using OrthoVenn2.^13^

### Phylogenetic analysis

The utilizes progressive Mauve, which applies an anchored alignment algorithm, to identify Locally Collinear Blocks (LCBs) shared in the genome.^12^ The LCBs coexisting among all genomes will be extracted and trimmed by trimAl to screen out phylogenetically informative regions.^14^ The authors used the final alignment to build a maximum likelihood phylogenetic tree by IQtree2.^15^ The Interactive Tree of Life (https://itol.embl.de) was used for the manipulation and annotation of the phylogenetic tree. The five known global clades and ERG11 mutations are marked in the phylogenetic tree.

### Antifungal susceptibility testing

The in vitro susceptibility of the isolate was determined using the Sensititre YeastOne colorimetric microdilution method (Thermo Fisher Scientific, Oxoid, USA) according to the manufacturer's instructions. *Candida krusei* ATCC6258 and *Candida parapsilosis* ATCC 22019 were used as the control isolates.^6^ Categorical results were obtained according to the following tentative MIC breakpoints for *C. auris* published by the CDC: fluconazole, 32 μg/mL; voriconazole, not available; amphotericin B, 2 μg/mL; caspofungin, 2 μg/mL; anidulafungin, 4 μg/mL; and micafungin, 4 μg/mL (https://www.cdc.gov/fungal/candida-auris/recommen-dations.html).

### Molecular docking analysis

To probe fluconazole interactions with the potential binding sites of *C. auris* ERG11, molecular docking between them was carried out using SYBYL X -1.2 software (Tripos Inc., St. Louis, MO, USA). The structures of drugs that were used to treat *C. auris* as CAM (Pubchem Compound ID: 84029) were retrieved from the NCBI PubChem database (https://pubchem.ncbi.nlm.nih.gov/) and were then prepared prior to docking.^16^ The energy minimization of compounds was carried out using the Tripos force field of SYBYL X -1.2 software.

Currently, the PDB database does not have the ERG11 protein structures of *C. auris* for reference. A BLASTp search against the PDB database showed that the protein has 70.2% identity with the protein lanosterol C14 alpha demethylase (PDB ID: 5V5Z) from *Candida albicans*. Hence, 5V5Z was selected as a template for tertiary structure prediction. The Protein Data Bank (PDB) database (https://www.rcsb.org/) was used to obtain the complete structure of 5V5Z. The template and ERG11 sequence were submitted to I-TASSER, an online server for modeling, and the best model was selected on the basis of higher C-score and lowest Z-score.^17^^,^^18^ The model was energy-minimized using SYBYL X-1.2. The model was further assessed using PROCHECK and ERRAT through the SAVES server (http://services.mbi.ucla.edu/SAVES/).^19^, ^20^, ^21^ Mutagenesis was achieved using PyMOL, and the structure was also subjected to refinement procedures similar to the wild-type structure.^22^

All the following operations were performed in the SYBYL X-1.2 software package. Hydrogen atoms and electric charges were added to the constructed target protein structure to repair the missing amino acid residues. The protein model was first optimized in the AMBER FF99 force field for 1000 iterations by steepest descent and then optimized to a convergence gradient of 0.05 kcal/(Å moL) by a conjugated gradient. The “Multi-Channel Surface” option of the Protomol Generations module was adopted to determine the active pocket. The Gasteiger-Hückel method was used to charge ligand molecules and convert them into three-dimensional structures. The optimized structure was docked with the Surflex-Dock module, and the interaction force between the ligands and proteins was calculated by the total score.^23^

## Results

### The genomic characteristics of strain CA01

To reveal the genomic properties of the CA01 *C. auris* strain, the authors performed Whole-Genome Sequencing assays that were depicted based on assembled genome sequences and prediction results of protein-encoding genes (Fig. S1). The outermost circle is the position coordinates of the genome sequence. A summary of the strain genomic features is given in Table 1. The genome size of CA01 was 12.82 Mb, and the GC content was 46.83%. The genome of the CA01 assembly included 4,473 genes, with a length of 6,593,280 bp and an average length of 1,474 bp. The internal gene length was 6,228,520 bp, with a gene internal GC content of 43.45%. The authors further performed analyses targeting repetitive sequences, including Long Terminal Repeat (LTR), DNA, Long Interspersed Nuclear Element (LINE), Short Interspersed Nuclear Element (SINE), and Rolling Circle (RC) repeat sequences (Table 2). The results showed that LTR was the most abundant type of repeat present in strain CA01, accounting for 43.89%, and the average length of LTR was 70 bp. The average lengths of DNA and LINE individually were 79 bp and 87 bp, respectively, and the percentage of the genome accounted for 25.6% and 30.63%, respectively. SINE and RC are less frequent, accounting for less than 2%.

**Table 1:** Genome assembly information of strain CA01.

Name	Value
Genome Size (bp)	12,821,800
Contigs	19
Gene Number	4,473
Gene Length (bp)	6,593,280
GC Content	46.83%
% of Genome (Genes)	51.42
Gene Average Length (bp)	1474
Gene Internal Length (bp)	6,228,520
Gene Internal GC Content	43.45%
% of Genome (Internal)	48.58

**Table 2:** Repetitive sequences of strain CA01.

Classes of repeats	Number	Total Length (bp)	Average length (bp)	Percentage of genome (%)
LTR	827	56277	70	0.4389
DNA	438	32824	79	0.256
LINE	484	39277	87	0.3063
SINE	31	1670	60	0.013
RC	23	1807	79	0.0141
Unknown	4	367	92	0.0029
Total	1807	125803	77	0.9812

LTR, Long Terminal Repeat; LINE, Long Interspersed Nuclear Element; SINE, Short Interspersed Nuclear Element; RC, Rolling Circle.

### Phylogenetic analysis of *C. auris*

**Fig. 1:**
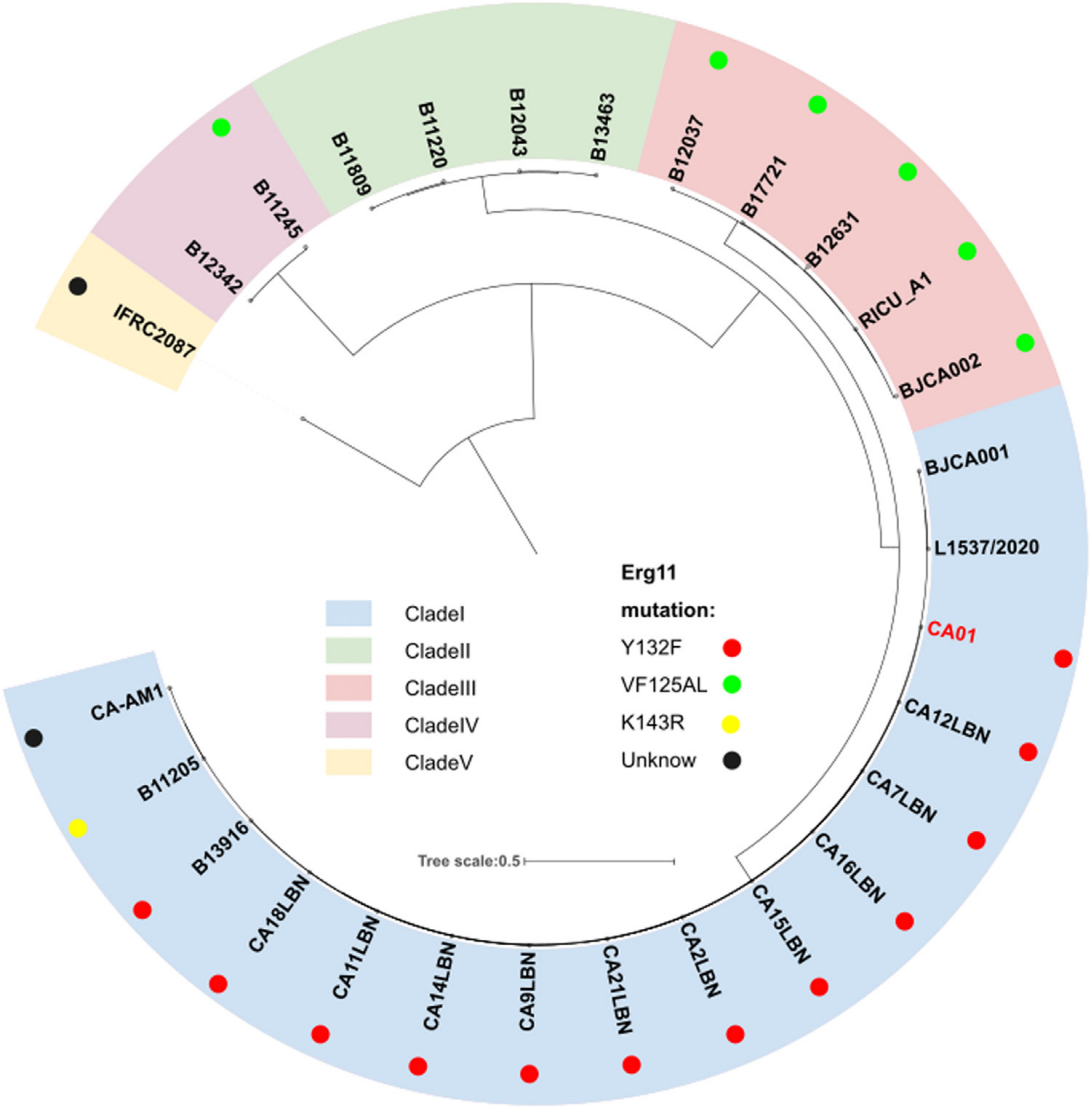
Phylogeny of *C. auris* genome strain, including CA01 from the present study and 27 publicly available from the NCBI database. The perimeter represents the five clades geographic distribution: clade I (blue), clade II (green), clade III (pink), clade IV (purple), and clade V (yellow). The dots represent different mutations.

To investigate genomic changes that could explain the emergence and different phenotypes observed between *C. auris* clades, CA01 was compared to all available *C. auris* genomes that had a strong phylogeographic structure comprising five distinct clades, including clade I (South Asia), clade II (East Asia), clade III (Africa), clade IV (South America) and clade V (Iran). Phylogeny analysis using the NCBI database demonstrated that strain CA01 belonged to the South Asia Clade (I), with the Y132F mutation (Fig. 1).

### Comparison of the antifungal susceptibility test

To examine the resistance levels to the available drugs, the authors tested the antifungal susceptibility of CA01 to nine different drugs (Table 3). The results exhibited a high Minimum Inhibitory Concentration (MIC) of fluconazole of CA01 of 256 mg/L, which was higher than the reports of BJCA001 and BJCA002. The MICs of the remaining eight drugs of strain CA01, such as anidulafungin, micafungin, caspofungin, 5-flucytosine, itraconazole, posaconazole, voriconazole, itraconazole, and amphotericin B, were equal to or less than 2 mg/L (Table 3), which is similar to the reports of BJCA001 and BJCA002. Collectively, the CA01 strain had relatively higher MICs for the tested drugs compared to the report of BJCA001, which showed a multidrug-resistant antibiotype and reduced bacterial susceptibility, especially for fluconazole. Furthermore, CA01 was resistant to fluconazole at a high level, owing to mutations in A395T of ERG11 that corresponded to the Y132F amino acid substitution site.

**Table 3:** Antifungal susceptibility testing of *C. auris* strain.

	AFG	MFG	CAS	5-FC	POS	VRC	ITC	FLC	AMB
**CA01**	0.12	0.12	0.12	0.12	0.06	1	0.12	256	2

MICs (Minimal Inhibitory Concentrations, mg/L) are shown. AFG, Anidulafungin; MFG, Micafungin; CAS, Caspofungin; 5-FC, 5-Flucytosine; POS, Posaconazole; VRC, Voriconazole; ITC, Itraconazole; FLC, Fluconazole; AMB, Amphotericin B.

### *Mutations of the C. auris* ERG11 *gene and molecular docking*

According to the four models generated, ERG11_Model_1 (Fig. S2A), whose exp. TM-score and exp. RMSD were 0.98±0.05 and 3.5±2.4 Å, respectively, had the best C-score (1.89) and were selected for molecular docking. The average, Root Mean Square (RMS), and distribution of Z-scores determined for ERG11 are shown in Fig. S2B. A Ramachandran plot showed that 83.5% of all residues are located in the most favored regions, 13.9% are in additionally allowed regions and 0.9% are in generously allowed regions (Fig. S2C). ERRAT produced an overall quality factor of 92.2 for ERG11 (Fig. S2D). The results suggested that the established model of ERG11 could be used for further studies.

**Fig. 2:**
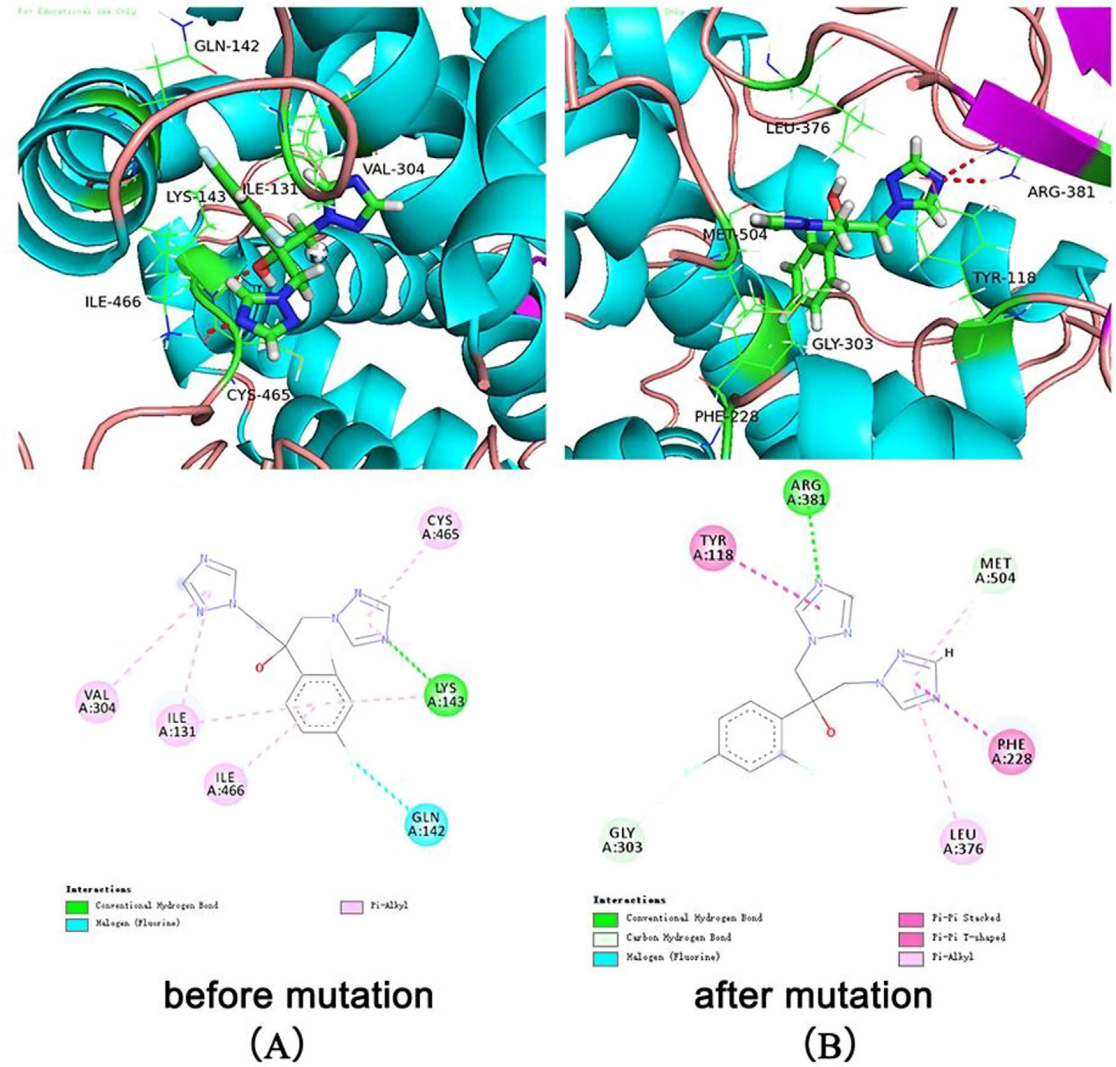
illustrates the interaction mode between fluconazole and ERG11. (A) The interaction between fluconazole and ERG11 before mutation. (B) The interaction between fluconazole and ERG11 after the A395T mutation.

The docking results showed that the native protein exhibited a lower binding energy than the mutant protein. The binding score of fluconazole with the native protein was 5.38, while it was 4.68 with the mutant protein (Table 4). The interaction mode between fluconazole and ERG11 is depicted in Fig. 2. In the left panel, fluconazole forms hydrogen bonds with LYS143 of the premutation ERG11 receptor and halogen bonds (fluorine bonds) with GLN142. In the right panel (post-mutation), fluconazole forms hydrogen bonds with ARG381 of the mutant receptor protein and π-π stacking hydrophobic interactions with PHE228 and TYR118. In summary, the molecular docking results indicate that the Y132F gene mutation can reduce the affinity of fluconazole toward its receptor, which could be one of the molecular mechanisms leading to fluconazole resistance. This provides a theoretical basis for the mechanistic study of FKZ resistance and the rational use of drugs in clinical settings.

**Table 4:** Binding evaluation parameters of ERG11.

Name	Total Score	Crash^a^	Polar^b^
ERG11	5.375	-1.0994	2.1025
ERG11 after the A293T mutation	4.6798	-0.8205	1.9214

a Crash represents the degree of unnecessary collisions caused by the ligand in the protein and self-clash between ligand atoms that are separated by rotatable bonds. Te nearer Crash value to zero, the better the binding mode is. Negative values represent penetration.

b Polar represents the contribution of the polar interactions to the total score.

### Comparative analysis of the genomic characteristics of strains CA01, BJCA001 and BJCA002

**Fig. 3:**
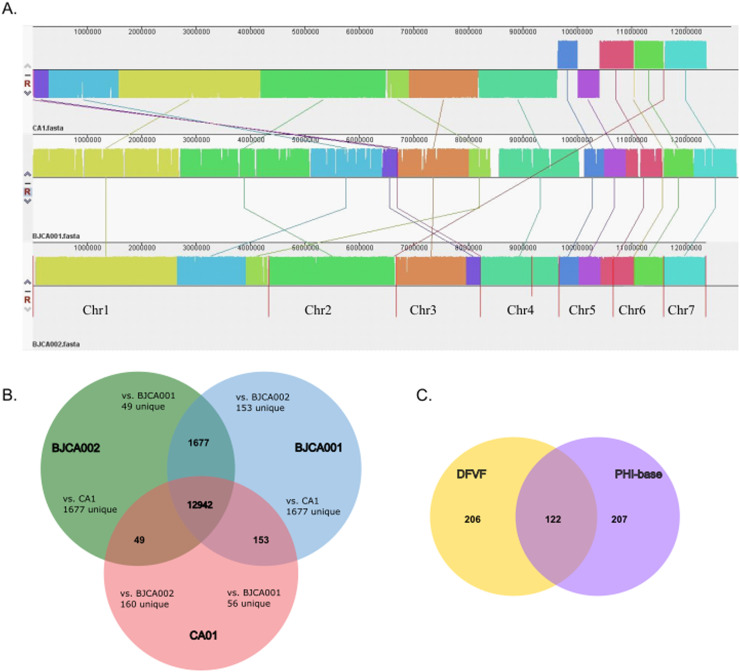
Comparative analysis of the genomic characteristics of strains CA01, BJCA001 and BJCA002. (A) Genome-wide gene synteny among CA01, BJCA001 and BJCA002. The authors identified orthologs between these isolates and then plotted chains of orthologs. Isolate names are shown to the left of their genomes, with vertical lines indicating scaffold borders. (B) Venn diagram of gene numbers in the genomes of the three strains. (C) Venn diagram of virulence genes shared in the genomes of the three strains.

To uncover the genetic basis of the difference in gene function of strain CA01, the authors elucidated gene function annotations using various databases. Consistent with the phylogenies of these isolates, the assemblies of the CA01 and BJCA001 chromosomes showed high similarity, and the two genomes were highly syntenic but not to BJCA002 (Fig. 3A). While the genomes of *C. auris* CA01 and BJCA001 are highly syntenic, the authors found evidence of a few large chromosomal rearrangements. For example, a large chromosomal rearrangement between chromosome 1 of strain CA01 and chromosome 2 of BJCA001 involved a translocation of two fragments of 2.7 Mb and 0.3 Mb, respectively.

Additionally, the authors searched for the unique genes and shared genes of the three species of *C. auris* through Venn diagram analysis, and all three shared 12942 genes. CA01 showed 56 and 160 unique genes when compared with BJCA001 and BJCA002, respectively (Fig. 3B). In addition, the virulence genes of three *C. auris* strains were analyzed and showed 122 shared genes after comparison between the DFVF database and PHI-base; 206 genes were unique to the DFVF database, and 207 genes were unique to PHI-BASE (Fig. 3C).

### Functional annotation analysis of virulence genes of strains CA01, BJCA001 and BJCA002

**Fig. 4:**
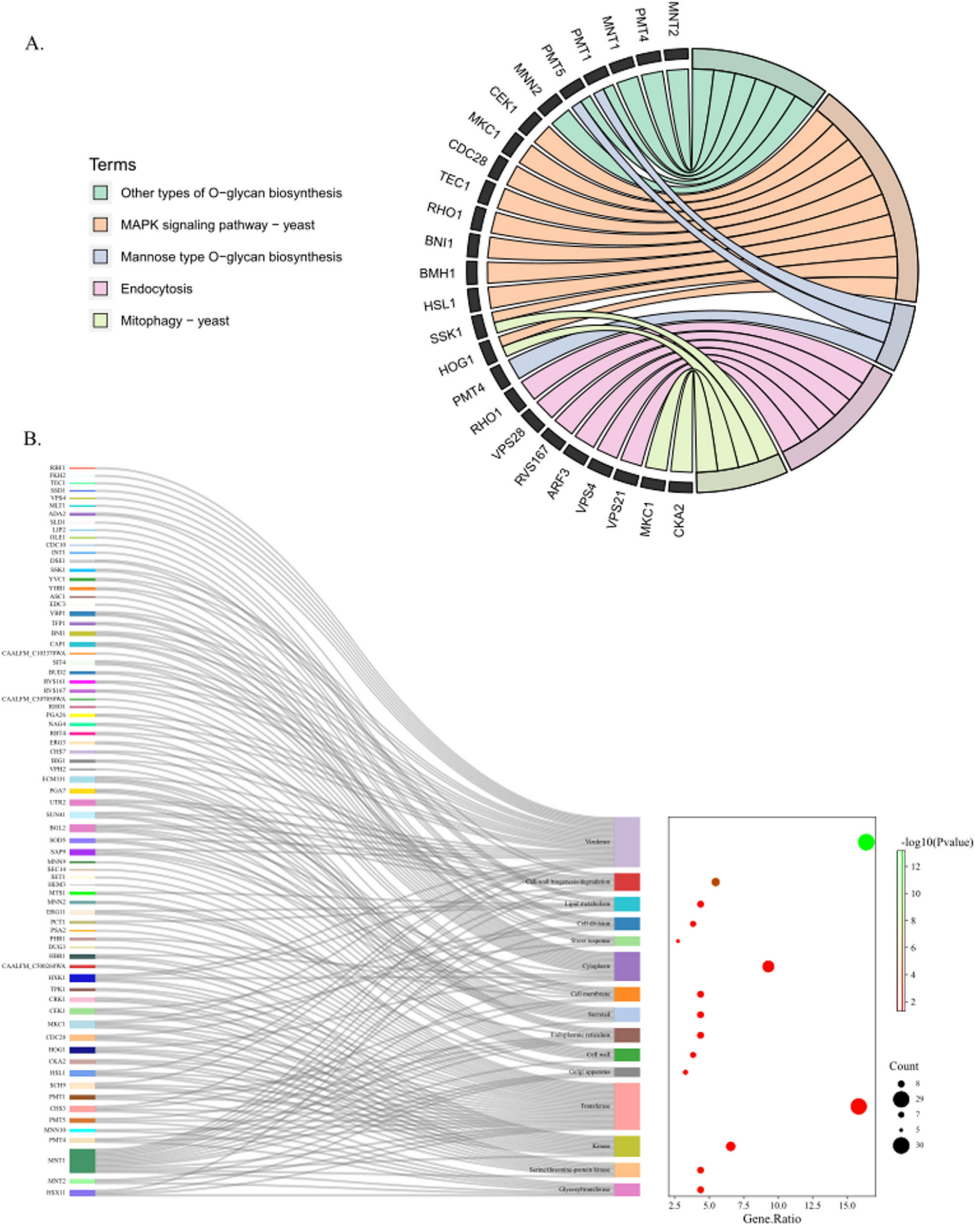
Annotation function analysis of virulence genes of strains CA01, BJCA001 and BJCA002. (A) KEGG classification of virulence gene function annotation. (B) GO classification of virulence gene function annotation.

To understand pathogenic processes and regulatory mechanisms, the authors further analyzed the function of virulence genes of three *C. auris* by KEGG and GO databases to obtain annotation information (Fig. 4A‒B). A total of 5 pathways were enriched, including the MAPK signaling pathway, O-glycan biosynthesis, endocytosis, mitophagy and mannose type O-glycan biosynthesis, in which 10, 6, 6, 4 and 3 genes were enriched, respectively. The MAPK signaling pathway contains the largest number of virulence genes, including CEK1, MKC1, CDC28, TEC1, RHO1, BNI1, BMH1, HSL1, SSK1, and HOG1. The mitophagy signaling pathway contains not only SSK1 and HOG1 but also MKC1 and CKA2. O-glycan biosynthesis-related genes include MNT1, MNT2, PMT1, PMT4, PMT5 and MNN2. Endocytosis relates to VPS family genes (VPS4, VPS8, VPS21), RHO1, RVS167 and ARF3. Furthermore, GO function annotations of virulence genes showed that 15 GO terms were enriched, in which virulence, transferase, and cytoplasm were the top three categories, including RBF1, FKH2, TEC1, SSD1, and VPS4.

## Discussion

This study investigated the genome characteristics and phenotypes of *C. auris* isolated from Beijing, China. The present findings revealed that strain CA01 belongs to the South Asia Clade (I) and carries mutations in A395T. Moreover, strain CA01 showed differences in antifungal susceptibility and genomic characteristics compared with the previously reported isolate of *C. auris* BJCA002 but was close to BJCA001. In addition, the CA01 strain exhibited a high level of resistance to fluconazole, and molecular docking analysis suggested that the A395T mutation is one of the contributing factors to this resistance. The present results found several putative genes coding for virulence factors in three *C. auris strains*, such as BMH1, BNI1, RBF1, and FKH2. This study not only expanded the species of *C. auris* at South Asia Clade (I) but also provides evidence-based clues of molecular features and virulence at the level of genes to develop novel diagnostics and therapeutic targets for *C. auris*.

*Candida auris* is an emerging multidrug-resistant yeast that can rapidly spread globally, causing invasive infections in humans with a high mortality rate.^2^ The main reasons for infections and outbreaks of *C. auris* in nosocomial settings are multidrug resistance and various virulence factors due to different genetic backgrounds.^24^ Therefore, the authors comprehensively analyzed the antifungal susceptibility and genome characteristics of strain CA01 by a comparative genomic analysis with the previously reported genomes of BJCA001 and BJCA002. The present results found that CA01 exhibited a high MIC of fluconazole that was 256 mg/L, higher than that of BJCA001 and BJCA002, which was probably related to a mutation of Y132F in the ERG11 gene of *C. auris*. Subsequently, the authors performed homology modeling and molecular docking to systematically investigate the binding mode of fluconazole with the wild-type and mutated target ERG11, aiming to uncover the resistance mechanism. The docking results revealed that the mutation Candida auris (ERG11:Y132F) resulted in geometric changes in the active pocket where FZC binds, leading to weakened binding affinity due to altered interactions and important residue modifications. A comparative analysis showed that FZC formed interactions, including hydrogen bonds and hydrophobic interactions, with amino acid residues such as ILE131, GLN142, LYS143, VAL304, CYS465, and ILE466 in the premutation ERG11 receptor, while the interaction with GLN142 formed a halogen bond (fluorine bond). However, with the mutated ERG11 receptor, FZC exhibited interactions with amino acid residues TYR118, LEU376, PHE228, MET504, and ARG381. This finding is consistent with previously published research. Previous studies reported that mutations in *C. auris* ERG11 significantly contribute to fluconazole and voriconazole resistance.^25^, ^26^, ^27^ Two ICU patients from southern Nigeria presented genomic analysis and found a confirmed mutation (ERG11:Y132F) that conferred drug resistance to azoles.^28^ Overall, through the systematic investigation of gene features, antifungal susceptibility tests, and molecular docking of FZC with CA01, this study provides a theoretical basis for clinical personalized medication and the design of novel drugs to overcome resistance.

In addition, the present results found that the genomes of CA01 shared the closest relationship with BJCA001, which was similar to BJCA001 in geographic origin, which belongs to the South Asia Clade (I). BJCA001 was separated from the Bronchoalveolar Lavage Fluid (BALF) of a hospitalized woman and identified higher invasiveness and virulence of the pathogen than BJCA002 strains.^6^ The previous findings indicated that CA01 is more likely to have similar higher virulence because of its closest relationship with BJCA001. Virulence factors can be maintained within the genome on pathogenicity islands and contribute to the pathogenicity of an organism.^29^ To uncover the virulence mechanisms of the three *C. auris strains* in the genome, the authors analyzed the function of virulence genes by databases, and genome sequencing showed that the MAPK signaling pathway, O-glycan biosynthesis, endocytosis, and mitophagy are among the most relevant molecular mechanisms of virulence traits. These results found that the majority of the MAPK pathway-related genes (CEK1, MKC1, CDC28, TEC1, RHO1, HSL1), O-glycan biosynthesis (MNT2, PMT4), endocytosis (ARF3, VPS21) and mitophagy-related genes (MKC1, CKA2, SSK1, HOG1) were involved in virulence, which is similar to a previous study. Many related studies have reported that the above gene is essential for adhesion, invasion, hyphal formation, biofilm formation, and virulence of fungal pathogens.^30^, ^31^, ^32^ For example, a lack of the Cek1- and Hog1-mediated pathways leads to a lethal phenotype in *Candida albicans*.^33^ In addition, previous studies revealed that the genes BMH1 and BNI1 in *C. albicans* were essential for cell cycle and morphogenesis growth^34^^,^^35^ or mediated the actin nucleation mechanism and impact on the differentiation of *C. albicans*,^36^^,^^37^ and the authors found a novel function of BMH1 and BNI1 that was involved in MAPK cascade signaling on the virulence attributes of *C. auris*. Moreover, the GO annotation findings indicate that the developmental regulators RBF1 and FKH2 are related to virulence in *C. auris*, as has been reported previously only in *C. albicans* or *Drosophila melanogaster* homologs. Fkh2 regulates *C. albicans* pathogenesis by cell cycle-independent phospho-regulation during hyphal growth.^38^ RBF1, a developmentally regulated gene with tissue-specific function, regulates transcription for Drosophila innate immunity.^39^ Overall, the authors summarized the associations of virulence-related genes of strains CA01, BJCA001, and BJCA002. The present results found several putative genes coding for core virulence factors in *C. auris*, such as BMH1, BNI1, RBF1 and FKH2.

## Conclusions

Herein, the authors present the genome results of a new *C. auris* CA01 and aimed to explore the genetics and phenotype of *C. auris*. Meanwhile, molecular docking and functional annotation analyses were utilized to investigate the drug resistance and pathogenicity of CA01 at the molecular level. The present findings expand current knowledge on the genomic characteristics, resistance traits, and virulence characteristics of *C. auris* and contribute to the global effort to address emerging fungal threats.

## Institutional review board statement

This study was reviewed and approved by the Aerospace Center Hospital (n° JHYLS-2023–013). Informed consent was obtained from the subjects in this study. All methods were performed in accordance with the relevant guidelines and regulations.

## Data availability statement

This Whole Genome project has been deposited at DDBJ/ENA/GenBank under the accession JAUDZI000000000. The version described in this paper is version JAUDZI010000000.

## Authors’ contributions

Conceptualization: Jing-Xian Yang; Methodology: Guan-Nan Ma; Software: Guan-Nan Ma, Ya-Tong Li; Data curation: Guan-Nan Ma, Yu-Peng Shi; Investigation: Jing-Xian Yang; Validation: Jing-Xian Yang; Formal analysis: Guan-Nan Ma; Supervision: Guo-Wei Liang; Funding acquisition: Jing-Xian Yang; Visualization: Jing-Xian Yang; Project administration: Guo-Wei Liang; Resources: Jing-Xian Yang, Guo-Wei Liang; Writing-original draft: Ya-Tong Li, Yu-Peng Shi; Writing-review & editing: Guan-Nan Ma. All authors will be informed about each step of manuscript processing, including submission, revision, revision reminder, etc., via emails from the system or assigned Assistant Editor.

## Funding

This study was supported by Capital Health Research and Development of Special Fund, Beijing, China (Project number: 2020-4-6084).

## Conflicts of interest

The authors declare that they have no known competing financial interests or personal relationships that could have appeared to influence the work reported in this paper.

## Appendix Supplementary materials

Image, application 1
