# Supplementary material for: Resistance and virulence genes characteristic of a South Asia Clade (I) Candida auris strain isolated from blood in Beijing
Source: Clinics (Sao Paulo). 2024 Sep 15;79:100497. doi: 10.1016/j.clinsp.2024.100497 (PMC11419799; doi:10.1016/j.clinsp.2024.100497)

CLINICS-D-23-00786_Supplementary Material

**Figure Supplementary 1 Genomic structure of CA01 strain.** From the outside to the inside, the first circle is the GC content of the genome. The inward blue part indicates that the GC content of this region is lower than the average GC content of the whole genome, and the outer purple part is the opposite. And the higher the peak, the more significant the difference from the average GC content. The second circle is the genome GC skew value, the inward green part indicates that the content of G in this region is lower than the content of C, and the outer pink part is the opposite. The third circle is the gene density, such as the gene density of rRNA snRNA tRNA, the darker the color, the greater the gene density in the window. The fourth circle is chromosome duplication.


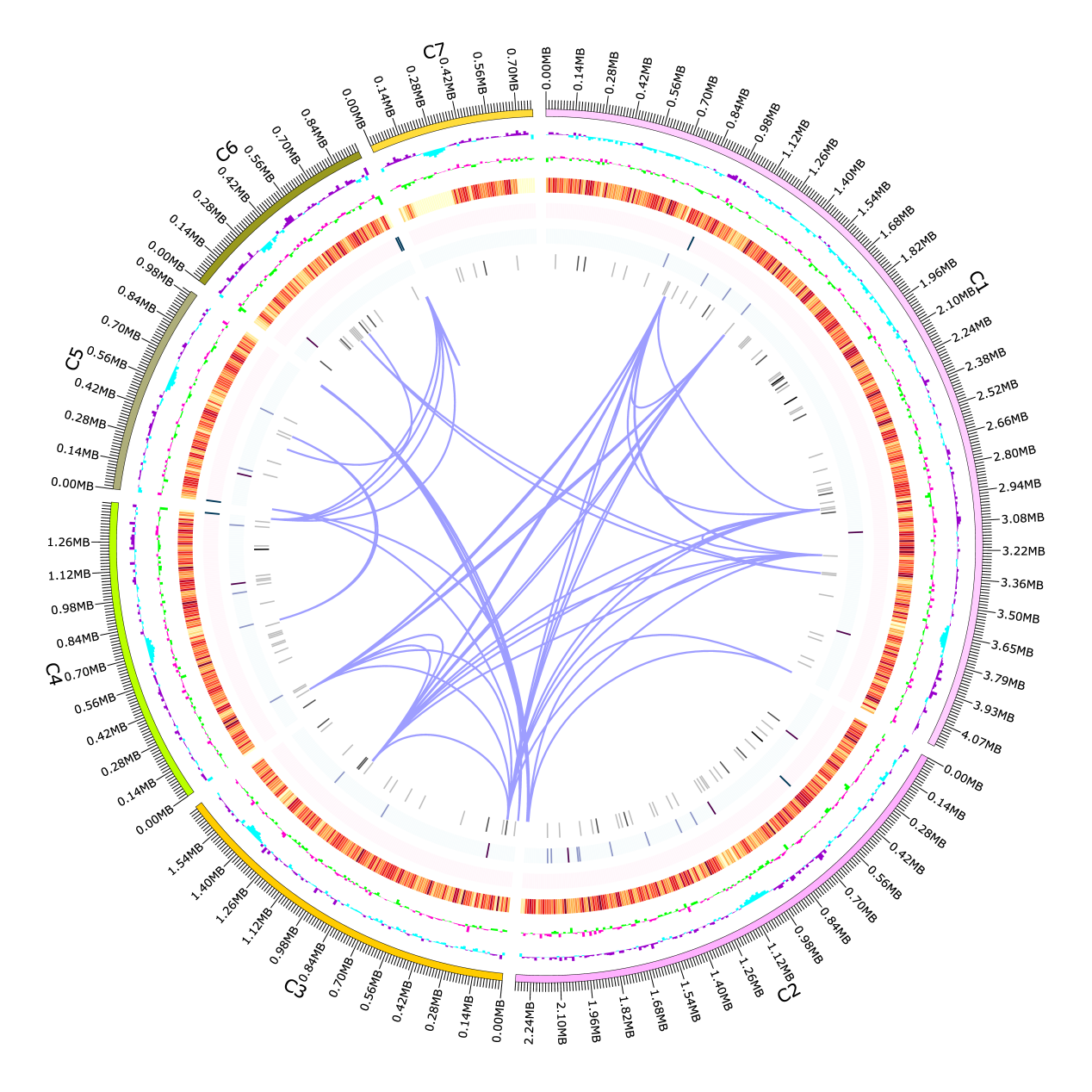


**Figure Supplementary 2 3D structural modeling of ERG11 and validation.** (A) ERG11_Model_1. (B) Ζ-score. (C) Ramachandran plot. (D) Error value.


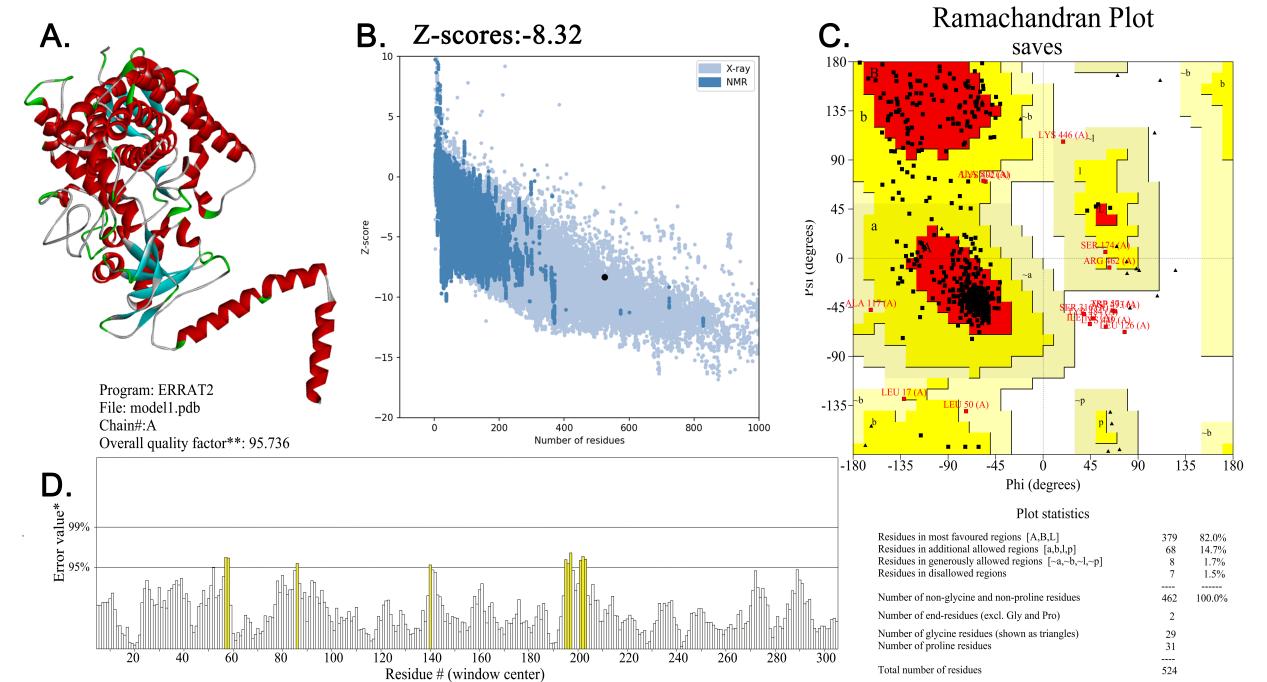

Supplement: Supplementary file 1 [file mmc1.docx]
